# DEqMS: A Method for Accurate Variance Estimation in Differential Protein Expression Analysis*[Fn FN2]

**DOI:** 10.1074/mcp.TIR119.001646

**Published:** 2020-03-23

**Authors:** Yafeng Zhu, Lukas M. Orre, Yan Zhou Tran, Georgios Mermelekas, Henrik J. Johansson, Alina Malyutina, Simon Anders, Janne Lehtiö

**Affiliations:** ‡ Department of Oncology-Pathology, Science for Life Laboratory, Karolinska Institutet, Stockholm, Sweden; § Institute for Molecular Medicine, University of Helsinki, Helsinki, Finland; ¶ Centre for Molecular Biology of Heidelberg University (ZMBH), Heidelberg, Germany

**Keywords:** Quantification, label-free quantification, statistics, data evaluation, mass spectrometry, quality control and metrics, differential analysis, FDR, TMT

## Abstract

Quantitative proteomics by mass spectrometry is widely used in biomarker research and basic biology research for investigation of phenotype level cellular events. Despite the wide application, the methodology for statistical analysis of differentially expressed proteins has not been unified. Various methods such as *t* test, linear model and mixed effect models are used to define changes in proteomics experiments. However, none of these methods consider the specific structure of MS-data. Choices between methods, often originally developed for other types of data, are based on compromises between features such as statistical power, general applicability and user friendliness. Furthermore, whether to include proteins identified with one peptide in statistical analysis of differential protein expression varies between studies. Here we present DEqMS, a robust statistical method developed specifically for differential protein expression analysis in mass spectrometry data. In all data sets investigated there is a clear dependence of variance on the number of PSMs or peptides used for protein quantification. DEqMS takes this feature into account when assessing differential protein expression. This allows for a more accurate data-dependent estimation of protein variance and inclusion of single peptide identifications without increasing false discoveries. The method was tested in several data sets including *E. coli* proteome spike-in data, using both label-free and TMT-labeled quantification. Compared with previous statistical methods used in quantitative proteomics, DEqMS showed consistently better accuracy in detecting altered protein levels compared with other statistical methods in both label-free and labeled quantitative proteomics data. DEqMS is available as an R package in Bioconductor.

## Abstract (precis)

Differential analysis of MS-data to identify biomarkers or to understand biology is a cornerstone in proteomics. DEqMS is a robust method for analysis of both labelled and label-free MS-data. The method takes into account the inherent dependence of protein variance on the number of PSMs or peptides used for quantification, thereby providing a more accurate variance estimation. Compared to current methods, DEqMS achieves better accuracy and statistical power in quantitative proteomics. The tool is available as user-friendly R package.

## Graphical Abstract

**Figure:**
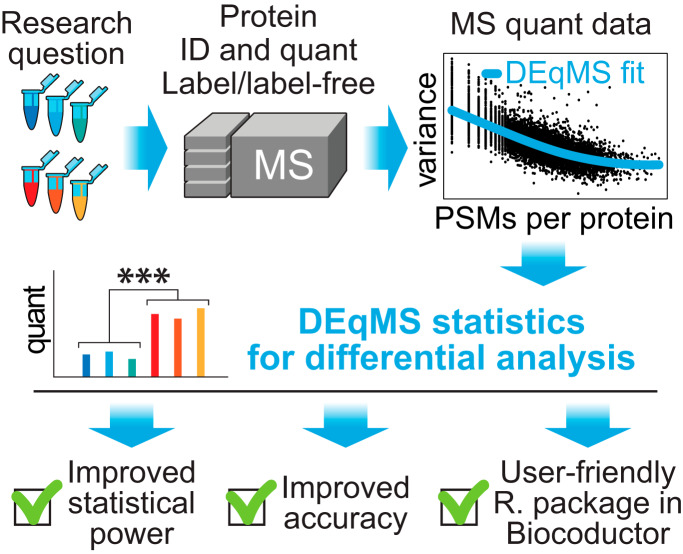

**Highlights**

- DEqMS is a method for statistical analysis of quantitative MS-data.
- Variance estimates based on the actual MS-data structure.
- Improved statistical power and accuracy in protein differential analysis.
- DEqMS is available as a user-friendly R package in Bioconductor.

Mass spectrometry (MS)-based proteomics is widely used for identification and quantification of proteins from complex biological samples (1). In a typical proteomics experiment, proteins are digested into peptides using a proteolytic enzyme, commonly trypsin, prior to MS-analysis. Peptides are usually fragmented multiple times, thus generating multiple peptide spectrum matches (PSMs)^1^ for the same peptide sequence. Peptide identification and quantification is subsequently performed in data analysis workflows using either label-free or labeled approaches. Both quantitative approaches rely on a hierarchical data structure: PSMs are nested into peptides which are then nested into proteins. Protein level quantification is subsequently generated through summarizing peptide or PSM level information.

In previous quantitative proteomics analysis, Student *t* test, ANOVA (2), Limma (3) and linear mixed models (2, 4–6), have been used to detect differentially expressed proteins (DEPs). In addition, other methods have been developed specifically for quantitative proteomics as exemplified by empirical Bayesian random censoring threshold model (EBRC) (7) and reproducibility-optimized test statistic (ROTS) (8). EBRC, developed for label-free MS-data, applies a random censoring threshold to achieve more accurate protein abundance estimates in the presence of missing values. ROTS is a modified form of t-statistics with a bootstrapping procedure to maximize the reproducibility of top ranked DEPs. In a previous analysis, ROTS was shown to outperform many statistical methods including Limma, *t* test, SAM and mixed models (9).

Limma, originally developed to analyze RNA microarray data, performs “moderated” ANOVA, *i.e.* it uses an empirical-Bayes approach to shrink gene-wise sample variance toward a common estimate based on all experimental data (3). In an evaluation performed by Kammers *et al.*, Limma detected more truly differentially expressed proteins compared with *t* test at the same false discovery rate (FDR) (10). Further, in a comparison between generalized linear model (GLM), mixed effects models and Limma in a tandem mass tag (TMT) 10-plex labeled proteomics data set, mixed effects models showed better specificity when combined with median sweeping normalization compared with the other two methods, whereas Limma had better statistical results independent of normalization methods (11).

However, there are problems in current methods. For proteins identified with a single PSM/peptide, the estimation of parameters in mixed effect models is less accurate, and in addition, it requires users to understand the correct random effects and to use appropriate statistical tests (11). In Kammers *et al.*'s study, proteins quantified with a single peptide were excluded from the Limma analysis (10), and D'Angelo *et al.* showed that omitting proteins quantified by a single peptide reduced false discovery findings (11). In a typical shotgun proteomics experiment, 10–20% of proteins are identified with only one peptide or PSM. Most of these are low abundant proteins that are potentially central in regulation of biological processes or contribute as biomarker candidates and are therefore important to keep in the analysis. Further, *t* test, ANOVA and Limma do not consider the difference in quantitative variation for proteins identified by different numbers of peptides/PSMs. It has previously been shown by us and others that the accuracy of protein abundance estimates varies by the number of peptides quantified (12–14). In microarray and RNA-seq data analysis, probe intensity and read counts are used to estimate the mean-variance relationship instead of using a common prior variance for all genes, resulting in improved performance over Limma (15, 16). Inspired by this approach, we have developed DEqMS (Differential Expression analysis of quantitative Mass Spectrometry data), to get a more accurate data-dependent estimation of protein variance based on the number of peptides or PSMs used for quantification.

## MATERIALS AND METHODS

### Experimental Design and Statistical Rationale

DEqMS is a statistical method for identification of differentially expressed proteins in MS-data, that takes into acount the dependence of variance on the number of PSMs or peptides used for protein quantification. The data sets used to evaluate the method and the statistics behind the method is presented in detail below.

### Data Set D1 - A431 Cells Treated with EGFR Inhibitor Gefitinib

Search results of this data set was downloaded from ProteomeXchange with identifier PXD006291 (17). PSM raw intensity table (combining all four IPG strips in the experiment) filtered at 1% PSM and protein level FDR (gene symbol centric search; 1,263,974 PSMs; 203,640 unique peptides; 10,166 genes), was used to calculate protein relative abundance by median sweeping method (18). First, raw intensity values are log2 transformed. Second, for each PSM, the median of log2 intensity is subtracted to get relative log2 ratio. Third, for each protein, its relative log2 ratio is calculated as the median of log2 ratio of all PSMs belonging to this protein. At last, the medians of protein log2 ratios in different samples are subtracted to get equal protein level in all samples, with the assumption that the treatment does not impact the total amount of proteins in different conditions. The generated protein table with log2 ratios without missing values (10,124 proteins) was used for *t* test, Limma, and DEqMS analysis. Biological triplicates of untreated and gefitinib treated (24 h) cells were used in the analysis for differential protein expression.

#### 

##### Data Set D2 - E. coli *Spike-in Label-free Data Set*

Search results of this data (50,016 unique peptides; 6566 proteins) were downloaded from the ProteomeXchange repository (PXD000279) (19). *E. coli* proteome was prepared into two conditions with 1:3 ratio (10 μg *versus* 30 μg) in triplicates and added into equal amount of human proteome background (50 μg Hela cells protein extract), see detailed sample preparation in (19). LFQ intensities in “proteinGroups.txt” table, for proteins with quant values in at least two samples per condition (5022 proteins), were log2 transformed and used as input matrix for all methods. To evaluate intensity-based Bayesian approach, trend = TRUE option was enabled in eBayes function provided in Limma. For variance stabilization normalization analysis, original protein LFQ intensity values were normalized by *justvsn* function in R package *vsn* before using Limma. ROC analysis was restricted to proteins unique to *E. coli* or human, with higher ratios in samples spiked-in with the higher amount of *E. coli* proteins.

### Data Set D3 - E. coli Spike-in TMT Data Set

#### 

##### Sample Preparation

One MCF-7 and one *E. coli* K-12 cell pellet were lysed and sonicated in a buffer containing 4% SDS, 25 mm HEPES pH 7.6 and 1 mm DTT. Total protein amount was estimated (DC protein assay, Bio-Rad, Hercules, California). Samples with different spiked in amounts of *E. coli* protein extract (3 replicates with 7.5 μg, 4 with 15 μg and 3 with 45 ug) in MCF-7 background (70 μg of protein extract) were prepared. Protein digestion (LysC and trypsin, sequencing grade modified, Thermo Scientific, Waltham, Massachusetts) was performed using a modified SP3-protocol (20). In brief, each sample was reduced with 1 mm DTT and alkylated with 40 mm Chloroacetamide. Sera-Mag SP3 bead mix (20 μl) was transferred into the protein sample together with 100% Acetonitrile to a final concentration of 70%. The mix was incubated under rotation at room temperature for 18 min. The mix was placed on the magnetic rack and the supernatant was discarded, followed by two washes with 70% ethanol and one with 100% acetonitrile. The beads-protein mixture was reconstituted in 100 μl LysC buffer (0.5 m Urea, 50 mm HEPES pH: 7.6 and 1:50 enzyme (LysC) to protein ratio) and incubated O/N. Finally, trypsin was added in 1:50 enzyme to protein ratio in 100 μl 50 mm HEPES pH 7.6 and incubated O/N. The peptides were eluted from the mixture after placing the mixture on a magnetic rack, followed by peptide concentration measurement (Bio-Rad DC Assay). Before labeling, samples were pH adjusted using TEAB pH 8.5 (50 mm final concentration). Thirty-five micrograms of each sample were labeled with an isobaric TMT-tag (Thermo Scientific). Labeling efficiency was determined by LC-MS/MS before pooling of samples. Sample clean-up was performed by solid phase extraction (SPE strata-X-C, Phenomenex, Torrance, California). Purified samples were dried in a SpeedVac.

##### High Resolution Isoelectric Focusing (HiRIEF)

After pooling and sample clean-up by solid phase extraction (SPE strata-X-C, Phenomenex), the sample pool were subjected to peptide IEF-IPG (isoelectric focusing by immobilized pH gradient) in pI range 3–10 (350 μg). Freeze dried peptide samples were dissolved in 250 μl rehydration solution containing 8 m urea and 1% IPG pharmalyte pH 3–10 and allowed to adsorb to the 24 cm linear gradient IPG strip by swelling overnight. Peptides were focused on the IPG strip as described in (21). After focusing, the peptides were passively eluted into 72 contiguous fractions with MilliQ water using an in-house constructed IPG extractor robotics (GE Healthcare Bio- Sciences AB, Uppsala, Sweden, prototype instrument) into a 96-well plate (V-bottom, Corning, Hickory, North Carolina product #3894), which were then dried in a SpeedVac. The resulting fractions were freeze dried and kept at −20 °C.

##### LC-MS/MS Analysis

Online LC-MS was performed using a hybrid Q-Exactive - HF mass spectrometer (Thermo Scientific). For each LC-MS/MS run, the auto sampler (Dionex UltiMate™ 3000 RSLCnano System, Thermo Scientific) dispensed 20 μl of solvent A to the well in the 96 V plate, mixed, and proceeded to inject 10 μl.

FTMS master scans with 70,000 resolution (and mass range 300–1700 *m*/*z*) were followed by data-dependent MS/MS (35,000 resolution) on the top 5 ions using higher energy collision dissociation (HCD) at 30–40% normalized collision energy. Precursors were isolated with a 2 *m*/*z* window. Automatic gain control (AGC) targets were 1e6 for MS1 and 1e5 for MS2. Maximum injection times were 100 ms for MS1 and 150–200 ms for MS2. The entire duty cycle lasted ∼2.5 s. Dynamic exclusion was used with 60 s duration. Precursors with unassigned charge state or charge state 1 were excluded. An underfill ratio of 1% was used.

Spectra data was converted to mzML files using ProteoWizard release: 3.0.10827 (2017–5-11) and searched with MS-GF+ (2016.10.26) (22) and Percolator (23). Precursor mass tolerance used was 10 ppm, fragment mass tolerance 0.11 Da, fixed modifications were TMT-10plex on lysines and peptide N termini, and carbamidomethylation on cysteine residues, oxidation on methionine was used as a variable modification. The protein database used for search was Uniprot (2018_04) human protein databases with *E. coli* protein database concatenated (78807 protein sequences) allowing for one tryptic miss-cleavage. PSMs and proteins were filtered at 1% FDR resulting in 308,001 PSMs; 122,235 unique peptides and 11,216 proteins.

The PSM table was aggregated into protein log2 ratio by median sweeping method described in the section of Data Set D1. The protein matrix of log2 ratios for proteins with no missing quant values (11,188 proteins) was used as input for subsequent *t* test, ROTS, Limma and DEqMS analysis. For Limma (vsn) method, PSM raw intensity table was first treated by variance stabilization normalization and then aggregated into protein ratio matrix. The statistical analysis was focused on the comparison between the 3 replicates with 7.5 μg and the 4 replicates with 15 μg of spiked in *E. coli* protein extract (the group with 45 μg spike-in had substantial global impact on protein ratios after normalization). The mass spectrometry proteomics data for data set D3 have been deposited to the ProteomeXchange Consortium via the JPOST partner repository with the data set identifier PXD013277.

##### Data Set D4 - U1810 Cells Treated by microRNA Mimics

Quantitative proteomics data of lung cancer cell line U1810 treated with miRNA mimics as previously described (24). Search results of the associated data (gene symbol centric search; PSMs and proteins filtered at 1% FDR; 369,273 PSMs; 107,509 unique peptides; 8,677 proteins) was downloaded from ProteomeXchange with identifier PXD004163. Same as data set D1, median sweeping method was used to summarize PSM level intensities to protein log2 ratio. The data was filtered to remove proteins with missing quant values (resulting in 8625 proteins), and analysis was performed using biological triplicates of miR-372 mimics and control siRNA treated cells.

##### Data Set D5 - Breast Cancer Cell Line Data Set

Quantitative proteomics data in triplicates of breast cancer cell lines (SKBR3, MCF7labA, MCF7labB, LCC2, HCC70, HCC1954, HCC1937, HCC1569, HCC1187, BT549, T47D, MDAMB157, CAL51, SUM149PT, HCC1143, BT20, HCC38, HCC1419) were generated and searched as for data set D3, except that MS data was searched gene centric and matched to human Ensembl version 75 (104,763 protein entries). A pool of all samples was used in one TMT tag as linker (denominator) between TMT sets. Labeling scheme can be found together with raw and search result data with identifier PXD013276. The intensity of internal reference (TMT tag 131) was used as denominator to calculate peptide ratios, the median of peptide ratios was taken as protein ratios. Protein ratios were then log2 transformed before DEqMS analysis. PSMs and proteins were filtered at 1% PSMs and protein level FDR resulting in 3,403,191 PSMs; 265,957 unique peptides and 11,408 proteins. Only proteins quantified in all TMT experiments were included in the analysis (9222 proteins). The minimum number of quantified PSMs across multiple TMT sets was used in DEqMS. The mass spectrometry proteomics data for data set D5 have been deposited to the ProteomeXchange Consortium via the JPOST partner repository with the data set identifier PXD013276.

##### Data Set D6 - Clinical Proteomics Human Brain

Search results of this data was downloaded from ProteomeXchange with identifier PXD006122 associated with a previous study (25). The mean intensity of two internal references (TMT tag 126 and 131) was used as denominator to calculate peptide ratios, the median of peptide ratios was taken as protein ratios. Protein ratios were then log2 transformed. PSMs and proteins were filtered at 1% PSMs and protein level FDR. The minimum number of quantified PSMs across multiple TMT sets was used in DEqMS. The cohort included samples from 8 Parkinson's disease dementia patients, 7 dementia with Lewy bodies patients, 9 patients with Alzheimer's disease and 8 elderly non-neurological controls.

##### Data set D7 - Phospho Proteomics

Search results of HeLa cells untreated (four biological replicates), treated with pervanadate or arrested in mitosis (three biological replicates each) were downloaded from ProteomeXchange with identifier PXD005410 (26). Same as data set D1, median sweeping method was used to summarize PSM level intensities to peptide log2 ratio. PSMs and peptides were filtered at 1% PSMs and peptide level FDR. The number of quantified PSMs per phosphorylated peptides was used in DEqMS.

The R scripts used to perform the analyses are provided as an R.markdown file (supplemental Data S1).

##### Statistical Methods

Log2 value of the relative intensity of protein *i* in sample *j* is denoted as *y_ij_*.

The way to calculate *y_ij_* for isobaric labeled and label-free data are described in the method section of data set D1 and data set D2.

In the following, we illustrate statistics calculations for the comparison of two groups for simplicity

##### Student t *Test*

Ordinary *t*-Statistics Is Calculated as

(1) $$t=\frac{\bar{Y_{G1}}-\bar{Y_{G2}}}{\sqrt{\frac{s_{1}^{2}}{n_{1}}+\frac{s_{2}^{2}}{n_{2}}}}$$

*n*_1_, *n*_2_ is the number of replicates in group 1 and group 2
*s*_1_, *s*_2_ is standard deviation in group 1 and 2
*G*1, *G*2 are the two groups
*Y*_*G*1_, *Y*_*G*2_ are the mean of two groups
  (2) $$s^{2}=\frac{\sum_{i\epsilon G}{\left(y_{ij}-\bar{Y_{G}}\right)}^{2}}{n-1}$$

##### ANOVA F-test

(3) $$F=\frac{n_{1}{\left(\bar{Y_{G1}}-\bar{Y}\right)}^{2}+n_{2}{\left(\bar{Y_{G2}}-\bar{Y}\right)}^{2}}{s_{p}^{2}}$$

(4) $$s_{p}^{2}=\frac{\left(n_{1}-1\right)s_{1}^{2}+\left(n_{2}-1\right)s_{2}^{2}}{n_{1}+n_{2}-2}$$

*Y* denotes the overall mean of the data

*s*_*p*_^2^ is pooled variance, which is a weighted within-group variance estimated using all samples in all groups.

##### Limma (Moderated t-*Statistics)*

(5) $$t=\frac{\bar{Y_{G1}}-\bar{Y_{G2}}}{s_{p\left[\text{moderated}\right]}\sqrt{\frac{1}{n_{1}}+\frac{1}{n_{2}}}}$$

*s*__*p*[*moderated*]__^2^ is posterior variance, a weighted average of pooled variance and prior variance. *s*__*p*[*moderated*]__^2^ is calculated as *fit$post.var* using eBayes() function from Limma package.

(6) $$s_{p\left[\text{moderated}\right]}^{2}=\frac{d_{p}s_{p}^{2}+d_{0}s_{0}^{2}}{d_{p}+d_{0}}$$

*s*_*p*_^2^ is pooled variance, *s*_0_^2^ is prior variance

*d*_0_ is the prior degrees of freedom, *d_p_* is the degree of freedom of the experiment

##### DEqMS Algorithm (spectraCounteBayes*) Explained Compared with Limma(trend* = *T)*

We want to emphasize here that DEqMS is developed on top of Limma. A core part of limma is its calculation of a so-called empirical-Bayesian prior variance, which describes one's prior expectation of the variance for a gene from the variance observed for all the other genes. By default, limma estimates a fixed prior distribution for all genes, but it has been shown by Sartor *et al.* (16) that it is advantageous to let the priors mean depend on further covariates, specifically on the genes' expression strength (averaged over all samples), if the variance seems to depend on it. Sartor *et al.* provided an R function, called “intensity-based moderated t-statistic” (IBMT), which could be used within an analysis with the limma package to modify limma's workflow and include such an intensity-dependent prior variance. A similar functionality, dubbed “limma-trend” was later added natively to limma by the limma package's authors (15).

In proteomics, a major determinant of quantification accuracy per sample, and therefore also of effective within-group variance, is the number of PSMs or peptides detected for a protein. We therefore modified the IBMT function of Sartor *et al.* (16) by changing the regression covariate from intensity to PSM or peptide count. In our DEqMS package, this modified IBMT function is called “spectraCounteBayes.” For TMT or iTRAQ labeled data, the number of PSMs is used to estimate prior variance as the quantification is done at MS2 level. For label-free data, the protein abundances are summarized from peptides and therefore the number of peptides per protein is used to estimate prior variance. The remainder of the package provides wrapper functions to conveniently tie in this function with the necessary calls to limma's functions.

For the reader's convenience, we provide in the following the mathematical details of the aprpoach. To this end, we follow closely the original exposition as given by Smyth (3), reproducing that publication's equations. We then show (in Equation (13)) the local (“loess”) regression on PSM count, which parallels the corresponding formula by Sartor *et al.* (16).

Definition of variables
logVAR: log transformed sample variance
*d*_0_: prior degree of freedom
*s*_0_^2^: prior variance
*d_g_*: degree of freedom of the experiment

*logVAR* is distributed as the sum of a constant and Fisher's Z distribution with the expected mean and variance calculated as:

(7) $$E\left(\log VAR\right)=\log s_{0}^{2}+\psi \left(d_{g}/2\right)-\psi \left(\frac{d_{0}}{2}\right)+\log\left(d_{0}/d_{g}\right)$$

(8) $$\mathrm{var}\left(\log VAR\right)=\psi \prime \left(d_{g}/2\right)-\psi \prime \left(d_{0}/2\right)$$

ψ and ψ′ are the digamma and trigamma functions

Define *e_g_* as the non-constant part of formula (7) for each gene after *s*_0_^2^ solved.

(9) $$e_{g}=\log VAR-\psi \left(d_{g}/2\right)+\log\left(d_{g}/2\right)$$

The expected mean of *e_g_*

(10) $$E\left(e_{g}\right)=\log s_{0}^{2}-\psi \left(d_{0}/2\right)+\log\left(d_{0}/2\right)$$

Based on this, *s*_0_^2^ can be calculated as

(11) $$s_{0}^{2}=e^{E\left(e_{g}\right)+\psi \left(d_{0}/2\right)-\log\left(d_{0}/2\right)}$$

Substituting *E*(*e_g_*) with the predicted *e_g_*, which is calculated as

(12) $$pred\left(e_{g}\right)=fitted\left(\log VAR\right)-\psi \left(d_{g}/2\right)+\log\left(d_{g}/2\right)$$

##### In DEqMS, *x* in equation 13 is defined as the log2 value of protein peptide or PSM count

(13) fitted(log⁡VAR)=loess(log⁡VAR∼x)$fitted

Based on formula (8), *d*_0_ can be estimated by solving

(14) $$\psi \prime \left(\frac{d_{0}}{2}\right)=\frac{1}{n}\sum {\left[e_{g}-pred\left(e_{g}\right)\right]}^{2}-\psi \prime \left(d_{g}/2\right)$$

ψ′(*y*) = *x*,*x* > 0 can be solved by deriving a Newton iteration of guaranteed and rapid convergence. Initial value of *d*_0_ is set to 0.1, by iterating *d*_0(*i*)_ = *i*/10. Convergence is reached when the value *diff* stops decreasing. *d*_0_ = *k*/10 when *diff* reaches its smallest value at k-th iteration.

(15) $$diff=\text{mean}{\left[e_{g}-pred\left(e_{g}\right)\right]}^{2}-\psi \prime \left(d_{g}/2\right)-\psi \prime \left(\frac{d_{0}}{2}\right)$$

##### In Limma (trend = T), *x* In Equation 13 Is Defined As the log2 Value of Protein Intensity

In analysis of proteomics data, the difference between Limma (trend = T) and DEqMS is what value logVAR is fitted against in formula (13).

Estimation of hyperparameters *s*_0_^2^ and *d*_0_ in DEqMS and Limma(trend = T) follows the same procedure as detailed above.

##### Multiple Testing Correction

All *p* values were corrected using Benjamini-Hochberg method (27) in DEqMS.

## RESULTS

### 

#### 

##### Dependence of Protein Variance On the Number of Quantified PSMs or Peptides

**Fig. 1.**
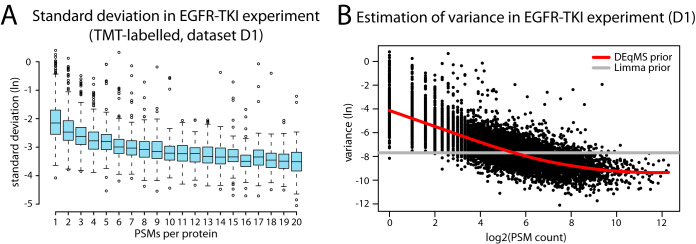
**Association between protein variance and the number of PSMs per protein used for quantification in MS proteomics data.** *A,* Boxplot showing the standard deviation of protein ratios within sample groups for proteins quantified with 1–20 PSMs in data set D1. *B*, Dependence of variance on the number of PSMs for all quantified proteins in data set D1. Gray and red curves indicate prior variance estimated by Limma and DEqMS respectively. Scatter points represent the pooled variance for individual proteins. PSM: peptide spectrum matches, (ln): natural logarithm.

To demonstrate the dependence of variance on the number of quantified PSMs we used an in-depth proteomics data set (TMT 10-plex labeled) of A431 cells sampled at different time points after treatment with the EGFR inhibitor gefitinib (17) (data set D1). As shown in Fig. 1*A*, for proteins quantified with 1–20 PSMs, the standard deviation (*s*) gradually decreases as the number of PSMs used for quantification increases. Similar dependence of variance (*s*^2^) on the number of quantified peptides or PSMs per protein is evident in other examples of label-free and labeled MS-data (supplemental Fig. S1*A*–S1*C*). Using a common prior variance on this data, Limma underestimates the variance for proteins quantified with few PSMs, and overestimates variance for proteins quantified with many PSMs (Fig. 1*B*). Therefore, the false positive rate for proteins quantified with few PSMs and the false negative rate for proteins quantified with many PSMs may increase. To reduce the false positive rate, proteins with a low number of PSMs/peptides are commonly removed from the analysis as discussed above. To salvage these proteins, and to correct the bias in the prior variance estimate, DEqMS estimates prior variances of proteins based on the number of quantified PSMs using non-parametric local regression (28) as shown in Fig. 1*B*. After performing the modified prior variance estimation, DEqMS uses Limma to perform the subsequent analysis steps, *i.e.* the moderated ANOVA calculation. With DEqMS, all proteins can be included in the statistical analysis with the number of PSMs/peptides used for quantification taken into account.

Subsequently, we investigated the impact on *p* values using DEqMS for DEP analysis between gefitinib treated cells and untreated controls (triplicates in both conditions), showing that lower *p* values were produced by DEqMS compared with Limma and *t* test (supplemental Fig. S2). In order to test if the increased sensitivity of DEqMS is accompanied with an increased false positive rate, we generated a simulated proteomics data set comprising nine samples and 6000 proteins (supplemental Fig. S3*A*). For each gene and each sample in the simulated data set, the log2 ratio was randomly sampled from a normal distribution. To keep the dependence of variance on PSM count like that observed in a real data set, the simulated data was generated separately for 6000 genes divided into 30 groups, quantified by 1–30 PSMs respectively. The number of genes in each group matches to that in a real data set (D4). The variance for genes in each group was determined by the fitted regression curve from the real data set D4 and the mean for all genes is centered at 0. The variance distribution of the simulation data mimics that of a real data set (supplemental Fig. S3*B*). Based on the simulated data we performed null comparisons by permutation of samples into two groups of three samples each, resulting in 84 possible comparisons without repetition. Importantly, this analysis showed that the false positive rate in DEqMS is slightly lower than that seen in Limma, whereas *t* test produce fewer false positives as expected, which is also evident form the *p* value distribution of the different methods (supplemental Fig. S3*C* and S3*D*).

##### Benchmarking of DEqMS in Label-free and Labeled Spike-in Data Sets

To systematically benchmark our method, we used two spike-in data sets (data set D2 and D3) for evaluation of the performance of DEqMS compared with Limma, *t* test, EBRC and ROTS. In addition, variance stabilization normalization (Limma(vsn)) (29) and intensity-based moderated *t* test (Limma(trend = T)) (16) were also included in the comparison as previous studies have shown that variance is dependent on peptide intensity (29).

**Fig. 2.**
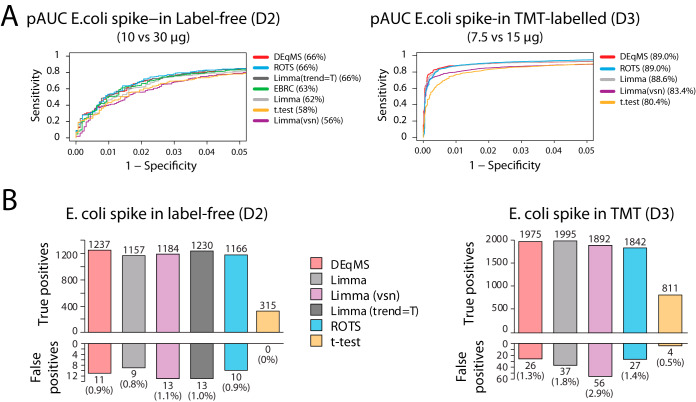
**Performance of DEqMS in spike-in MS proteomics data.** *A*, ROC curves for different methods used to analyze label-free (left) and TMT-labeled (right) *E. coli* spike-in data. Absolute value of t-statistics reported by different methods was used to compute area under curve. Indicated in the parentheses for each method is the partial area under curve (pAUC, in the range specificity>95%) expressed as a percentage relative to the maximum value in the specified range. Limma(vsn) shows the result of applying Limma on intensities normalized by variance stabilization method. *B*, True positive and false positive (adjusted *p* value<0.01) findings in label-free (left) and TMT-labeled (right) spike-in data using different analysis methods. Limma (trend = T) was not evaluated for TMT data because all other methods use protein log ratio matrix as input, and Limma (trend = T) requires estimation of protein intensity from PSM intensity, which is not a common practice to analyze TMT data. Associated data and results are available in supplemental Tables S1–S3.

In the label-free data set (D2), either 10 or 30 μg of *E. coli* protein extract was spiked into human protein extracts (50 μg) in triplicates (19). In the TMT 10plex-labeled data set (D3, supplemental Table S1), either 7.5 (3 replicates), 15 μg (4 replicates) or 45 μg (3 replicates) of *E. coli* peptides was spiked into 70 μg of human peptides. The analysis below is performed comparing samples spiked in with 7.5 μg and 15 μg of *E. coli* proteins. In the label-free and labeled experiments, 6566 (1902 *E. coli*, 4664 human) and 11,188 (2474 *E. coli*, 8764 human) proteins were identified and quantified, respectively. To evaluate the performance of different methods, receiver operating characteristic (ROC) curve analysis was used (Fig. 2*A*). Comparing partial area under curve (pAUC, 95% specificity), DEqMS performs better or at least as well as all other methods tested. Next, we applied a 1% FDR cutoff to investigate the number of significant genes reported by the different methods. In the label-free spike-in data set, EBRC failed to generate any significant findings after multiple testing correction despite having competitive result in the ROC analysis. Filtered by adjusted *p* value <0.01, DEqMS reported in total 1237 true differentially expressed *E. coli* proteins, and 11 falsely detected human proteins (Fig. 2*B*, supplemental Table S2). The second best method was Limma(trend = T), reporting 1230 true positives and 13 false positives. In comparison, variance stabilization normalization (vsn) did not improve the result. Although generating the same pAUC value, ROTS missed more true positives than DEqMS. *t* test detected the lowest number of true positives, which is not unexpected because of its low statistical power. In the TMT labeled data, median sweeping normalization, which showed better accuracy over quantitle normalization in a previous study^11^, was used to summarize the PSM intensity table to protein log2 ratio table. In our analysis, median sweeping normalization performed better when compared with variance stabilization normalization in Limma(vsn) (Fig. 2*B*, supplemental Table S3). Considering both the total number of significant findings and the percentage of false positives for each method, DEqMS performs better or as well as all other methods tested in both the label-free and the labeled data sets (Fig. 2*B*).

##### Application of DEqMS in Real World MS Proteomics Data

**Fig. 3.**
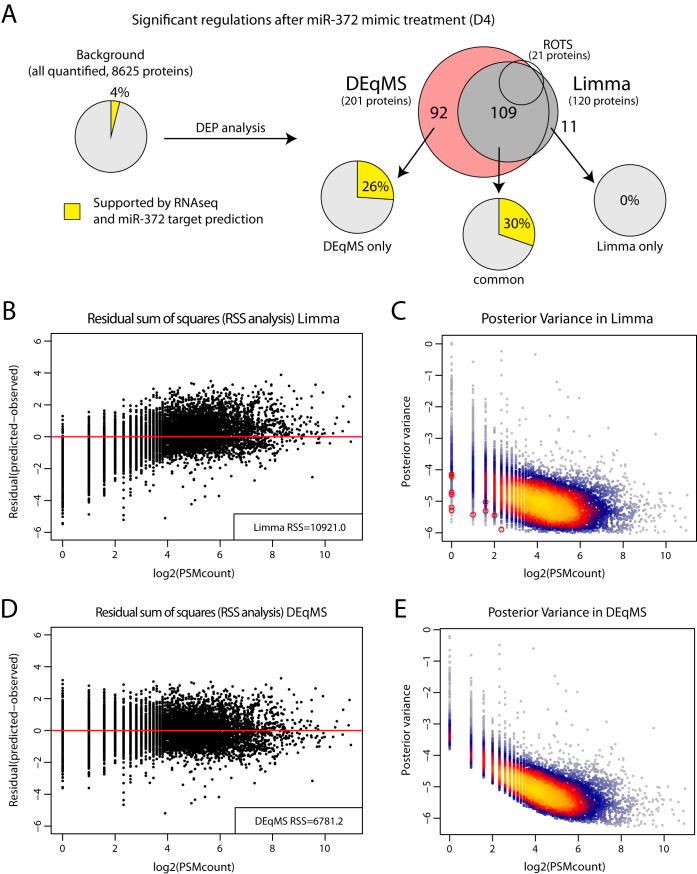
**Application of DEqMS in real world MS proteomics data.** a. Overlap of significant findings in the analysis of miR-372 mimic treated U1810 cells using different methods. Indicated in the figure is the number of significantly regulated proteins (DEPs) as identified by DEqMS only, Limma only, by both methods or by ROTS. Also indicated in piecharts for different sets of DEPs is the support of the corresponding mRNAs as miRNA targets. Support (yellow) indicates proportion of proteins where the corresponding mRNA was downregulated as evaluated by RNA-seq analysis, as well as predicted as being miR-372 targets by microRNA target prediction algorithms. For comparison, the overall background support in the entire data set is also shown. *B–E*, Comparison of variance estimate between Limma and DEqMS showing residual sum of squares (RSS) analysis of the prior variance estimate (*B*, *C*) and the relation between posterior variance and PSM count (*D*, *E*). The red circles in (*D*) indicate the 11 significant genes uniquely found by Limma, whereas these were rejected in DEqMS after adjusting the variance based on the number of quantified PSMs.

The two spike-in data sets (D2 and D3) represent simplified versions of normal quantitative proteomics data sets because all DEPs have equal, and quite large fold changes (2-fold or 3-fold in the tested labeled and label-free data set respectively), whereas there is no biological variation between replicate samples. To challenge the best performing methods further, we therefore applied them on a previously published data set where the impact of three different microRNA mimics on U1810 cells was investigated using quantitative proteomics and TMT 10-plex labeling (24). This data set (D4) is representative of an in-depth quantitative proteomics analysis with 8625 proteins (PSM and protein FDR<1%) quantified (see supplemental Fig. 4*A* for distribution of number of PSMs/protein). Here we focus on the comparison between cells treated with control siRNA and cells treated with miR-372 mimics, both present as triplicates in the proteomics data. The effects of microRNAs on protein levels are often relatively small, and the low number of expected direct targets of each microRNA makes it challenging to find biologically relevant significant hits. To evaluate the output of the DEP analysis, corresponding mRNA level analysis by RNA-Seq as well as microRNA target prediction algorithms were used as support for direct microRNA targets as previously described (24). Comparing miR-372 mimic treated cells and controls, *t* test identified a single significant DEP after correction for multiple testing (1% FDR), whereas 21 DEPs were identified using ROTS. DEqMS and Limma generated 201 and 120 DEPs respectively, with 109 DEPs in common (Fig. 3*A*, supplemental Fig. 4*B*). Importantly, both the 92 DEPs exclusively identified by DEqMS and the 109 DEPs identified by both methods were enriched in proteins where the corresponding mRNAs were downregulated and supported as direct miR-372 targets by prediction algorithms compared with the background (all proteins quantified in the experiment). In contrast, this was not true for the 11 proteins exclusively identified by Limma (Fig. 3*A*). To explain the improved performance of DEqMS over Limma, we plotted the residual sum of squares and the posterior variance of the two methods. This analysis shows that the error of the estimated protein variance in Limma is dependent on the number of PSMs used for quantification (Fig. 3*B*), whereas DEqMS was able to correct this bias (Fig. 3*C*). Consequently, this leads to differences between methods in posterior variance, where DEqMS eliminates false significant proteins quantified by one or two PSMs because of stochastic extreme low variance (Fig. 3*D*, 3*E*). In addition, DEqMS rescues many proteins quantified by multiple PSMs that were missed by Limma because of overestimated variance (supplemental Fig. S4*C*).

##### Using DEqMS in Data Sets with Multiple TMT Experiments

**Fig. 4.**
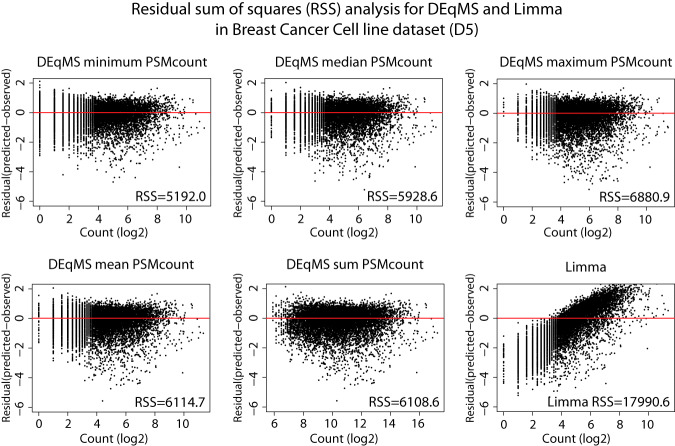
**Comparison of different PSM count metrics for data sets based on more than one TMT-experiment.** For data sets based on more than one TMT-experiment, proteins are usually quantified with a different number of PSMs in different TMT experiments. In DEqMS these numbers has to be unified to a single PSM count metric for each protein. The plots show the residual sum of squares (RSS) analysis for different PSMcount metrics. Shown in the figure are RSS plots for breast cancer cell line data set (D5) for DEqMS using minimum, median, maximum, mean and sum PSMcount and for Limma.

To investigate the performance of DEqMS in a larger data set based on multiple TMT experiments, we used a data set where 18 different breast cancer cell lines were analyzed in triplicates (D5, supplemental Table S4). In total six TMT-10plex experiments were performed to analyze the 54 samples as detailed in Materials and Methods. Importantly, replicate samples from each cell line were placed in three different TMT experiments to avoid confounding biological effects with batch effects. As more than one TMT-experiment was performed we first investigated what PSM metric to use for each protein when performing the DEqMS analysis; minimum, mean, median, sum or maximum number of PSMs/protein for quantificaiton across TMT-experiments. In all cases, the variance showed a dependence on the number of PSMs (supplemental Fig. S5). To identify the metric generating the best fit for prior variance we calculated the residual sum of squares (RSS) as a measure of the deviation between the predicted and the actual variance. This analysis showed that the best fit (smallest RSS) was produced when the minimum number of PSMs across experiments was used (Fig. 4). This result indicates that it is important to consider the weakest TMT-experiment for each protein, *i.e.* the one relying on the lowest number of PSMs, when performing DEqMS analysis in data sets consisting of multiple TMT experiments. As a comparison, RSS for Limma was also plotted, clearly indicating the bias in the estimated prior variance (Fig. 4).

**Fig. 5.**
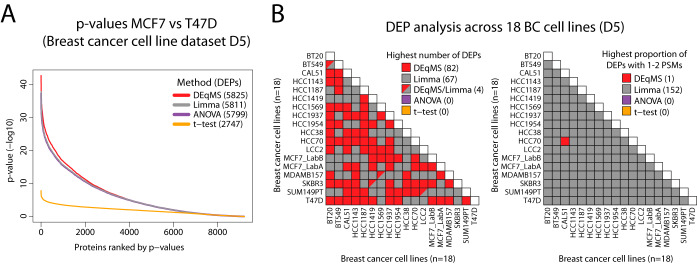
**Application DEqMS in a data set based on multiple TMT-experiments.** *A*, Distribution of *p* values (not adjusted) from statistical analysis using different methods for comparing breast cancer cell lines MCF7 and T47D. Indicated in parenthesis for each method are the number of DEPs identified with *p* value<0.01 after correcting for multiple testing by Benjamini-Hochberg method. *B*, Output from in total 153 different comparisons between breast cancer cell lines in data set D5. Left figure indicate for each comparison the method that identified the highest number of DEPs. Right figure indicate for each comparison the method that identified the highest proportion of DEPs with low (1–2) number of PSMs used for quantification.

For comparisons between methods, we used a common matrix design (with all 18 cell lines as one factor) when using DEqMS, Limma and ANOVA. As an example of the output of the analysis, comparing two of the luminal breast cancer cell lines (MCF7 and T47D) resulted in a large number of significant DEPs for DEqMS (5825) as well as Limma (5811, Fig. 5*A*). In this setting, ANOVA also generated a similar number of significant DEPs (5799), whereas *t* test generated much fewer significant DEPs (2747). Overall, the differences in the total number of DEPs identified were small between DEqMS, Limma and ANOVA. This is not a surprise because in larger MS-data sets with more samples (*i.e.* replicates and/or sample groups), the influence of the estimated prior variance on the posterior variance becomes smaller (see formula (6) in Materials and Methods) and the degree of variance shrinkage in Limma and DEqMS is decreased. Importantly, all these methods calculate pooled variances for each protein using all samples in all sample groups, whereas *t* test use only the samples in the two groups compared. Therefore, the statistical power is inferior in *t* test compared with the other methods, which is clearly indicated by the *p* values generated by the different methods (Fig. 5*A*, supplemental Fig. 6).

We then summarized the number of significant DEPs between all 18 cell lines and among them how many were quantified with 1–2 PSMs. DEqMS identified the highest number of DEPs in 82 of 153 comparisons, and Limma identified the highest number in 67 comparisons (Fig. 5*B* and supplemental Table S5). The major difference between the two methods was that Limma detected a higher proportion of DEPs quantified by 1–2 PSMs than DEqMS in all comparisons but one (Fig. 5*B*). Such consistent results emphasize the fact that DEqMS is a more stringent method when testing the significance of differential expression for proteins quantified by 1–2 PSMs.

##### DEqMS Application in Clinical Proteomics and PTM Analysis

In cell line studies as described above, the sample variance is relatively low compared with that in clinical samples. In order to demonstrate that the dependence of variance on the number of PSMs holds true also for clinical samples, we used a proteomics data set (D6, TMT-labeled) containing 32 post-mortem human brain samples from a study of dementia (25). Samples in this study included; Parkinson's disease with dementia; dementia with Lewy bodies; Alzheimer's disease; and older adults without dementia. As demonstrated in supplemental Fig. S7*A*, even though variance within sample groups in the clinical samples is much larger than that in cell line studies, we could still see that protein variance gradually decreases with an increasing number of PSMs used for quantification. This result indicates that the benefits of DEqMS would apply also in clinical proteomics data, however not as pronounced as in cell-line data.

Another quantitative proteomics field where large sample to sample variation makes statistical analysis challenging is post translational modification (PTM) analysis. The reason for this is that the analysis relies on quantification of specific modified peptides and almost all quantifications are therefore based on a single unique peptide. This results in high sample-to-sample variation in the quantitative analysis. To evaluate the dependence of variance on the number of PSMs in a typical PTM analysis, we analyzed an in-depth phospho-proteomics data set (D7) where samples were either untreated, treated with a tyrosine-phosphatase inhibitor or arrested in mitosis (TMT-labeled, four, three and three replicates respectively) (26). This analysis showed again a dependence, where variance decreased with increasing number of PSMs per phospho-site (supplemental Fig. S7*B*), indicating that DEqMS could improve the output of statistical analysis in quantitative PTM proteomics experiments.

## DISCUSSION

Until a few years ago, lack of convenient statistical tools impeded RNA- level differential expression analysis in the transcriptomics field for biologists with limited knowledge in statistics. Acknowledgment of this problem spawned research and development of methods by us (*DESeq (*30) and *DESeq2*(31)) and others (*edgeR*(32) and *Limma*(33, 33)). These methods are optimized for the specific transcriptomics data structure, to allow accurate and powerful analysis of significant differences in RNA levels. Today, such methods form a standardized backbone for analysis of transcriptomics data. Although MS-based methods have similarly become widely used for protein quantification, the statistical methods for quantitative proteomics analysis are less well established.

As has been noticed before and can be seen also from our results, Limma performs well for proteomics data even though it has been developed for expression microarrays. Importantly, we show here that the performance can be further improved by modifying Limma's variance prior estimation to consider the dependence of variance on the number of detected peptides/PSMs per protein. Our tool, DEqMS, performs this modified variance estimation (Fig. 1*B*), thus achieving better performance as shown in multiple data sets. In other words, DEqMS considers the actual MS-proteomics data structure to improve accuracy of DEP detection. Further, our method allows for inclusion of proteins quantified with low number of PSMs without increasing false positive rates, thus salvaging a large portion of low abundant proteins for the downstream analysis. This increases the chance of important biological findings. On all here tested data sets both label or label-free quantification, DEqMS consistently provides leading performance. The other two recently proposed method, EBRC and ROTS both have their own limitations. For instance, EBRC is developed only for label-free data, and whereas ROTS has competitive results, it is limited to two group comparisons only, lacking the flexibility to analyze multiple group experiments. Compared with Limma, DEqMS clearly performs better in small sample size experiments (shown in data set D4) because of improved protein variance estimate (Fig. 3). This advantage of DEqMS gradually decreased with increased sample size and sample variance (illustrated in data set D5, D6 and D7), but never results in inferior performance compared with Limma.

As an R package developed for quantitative proteomics analysis, DEqMS also provides functions (such as median sweeping normalization) to aggregate peptide or PSM intensities to protein abundance and auxiliary functions to visualize fitting curves and raw peptide intensities in different samples. Further, it is a computationally fast method as the analysis of 10,000 proteins finishes in a second with a standard PC. Taken together, DEqMS is a robust, universal and highly competitive statistical method for differentially expressed protein analysis of both labeled and label-free proteomics data.

DEqMS improves over current methods by more accurate protein variance estimation, thus achieving better accuracy and statistical power. The tool is available as user-friendly R package.

## DATA AVAILABILITY

Search result of previously published data set D1-D2 was downloaded from ProteomeXchange (http://www.proteomexchange.org/) with identifier PXD006291 and PXD000279. Data set D4 was downloaded with identifier PXD004163. Data set D6 and D7 were downloaded with identifier PXD013276 and PXD005410. The data set D3 and D5 generated by us have been uploaded to ProteomeXchange accessed through identifier PXD013277 and PXD013276.

DEqMS and an R. vignette describing the use of the package is available through Bioconductor (34) at https://bioconductor.org/packages/release/bioc/html/DEqMS.html. The package is also deposited at GitHub https://github.com/yafeng/DEqMS.

## Supplementary Material

Supplementary Figures

Supplementary Tables

Supplementary Data 1

## Footnotes

^1^ The abbreviations used are:

PSM: peptide spectrum matches
DEP: differentially expressed proteins
EBRC: empirical Bayesian random censoring threshold model
ROTS: reproducibility-optimized test statistic
TMT: tandem mass tag
DEqMS: Differential Expression analysis of quantitative Mass Spectrometry
HiRIEF: High Resolution Isoelectric Focusing
vsn: variance stabilization normalization.
