# Supplementary material for: DEqMS: A Method for Accurate Variance Estimation in Differential Protein Expression Analysis
Source: Mol Cell Proteomics. 2020 Mar 23;19(6):1047–57. doi: 10.1074/mcp.TIR119.001646 (PMC7261819; doi:10.1074/mcp.TIR119.001646)
Supplement: Supplementary Figures [file 153965_2_supp_496343_q7hy2c.pdf]

Supplementary material related to:

DEqMS: a method for accurate variance estimation in differential protein expression analysis

Yafeng Zhu*[1], Lukas M. Orre*[1], Yan Zhou Tran[1], Georgios Mermelekas[1], Henrik J. Johansson[1], Alina Malyutina[2], Simon Anders[3], Janne Lehtiö[1$].

[1]Department of Oncology-Pathology, Science for Life Laboratory, Karolinska Institutet, Stockholm, Sweden.
[2]Institute for Molecular Medicine, University of Helsinki
[3]Centre for Molecular Biology of Heidelberg University (ZMBH), Heidelberg, Germany.
* these authors contribute equally as first author.
[$]Correspondence should be addressed to janne.lehtio@ki.se

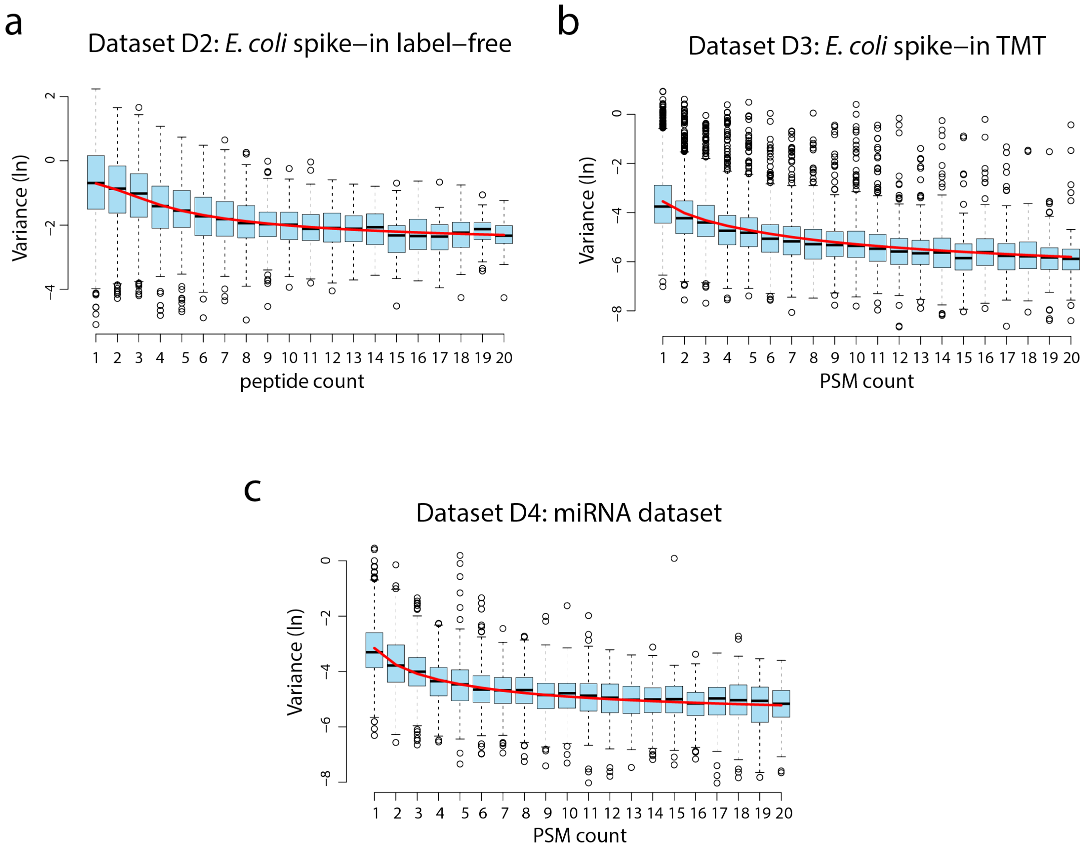

Supplementary Figure 1. Protein variance within sample groups in relation to the number of peptides or PSMs quantified per protein. Only proteins quantified with up to 20 peptides or PSMs are shown here. The red curve is fitted prior variance of the proteins in DEqMS. **a-c** shows: *E. coli* spike-in label-free dataset (D2), *E. coli* spike-in TMT labelled dataset (D3), the microRNA mimics treated U1810 cell dataset (D4). PSM: peptide spectrum matches, (ln): natural logarithm.

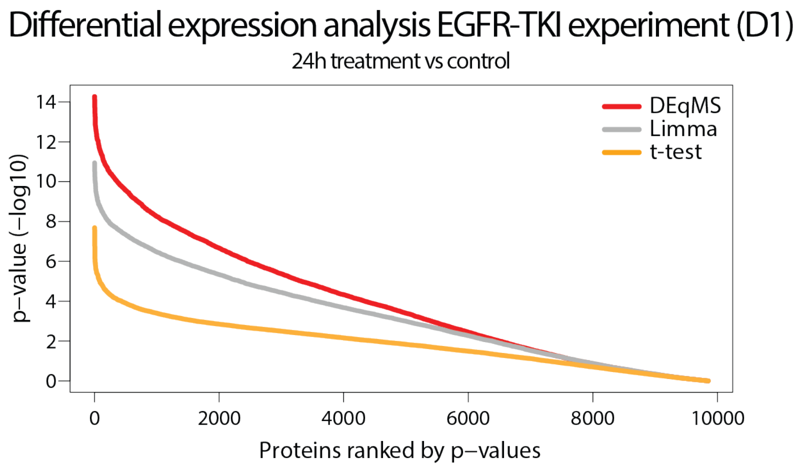

Supplementary Figure 2. Distribution of p-values (not adjusted) from statistical analysis using different methods for comparing gefitinib treated (24h) and untreated A431 cells (3 vs 3 replicates).

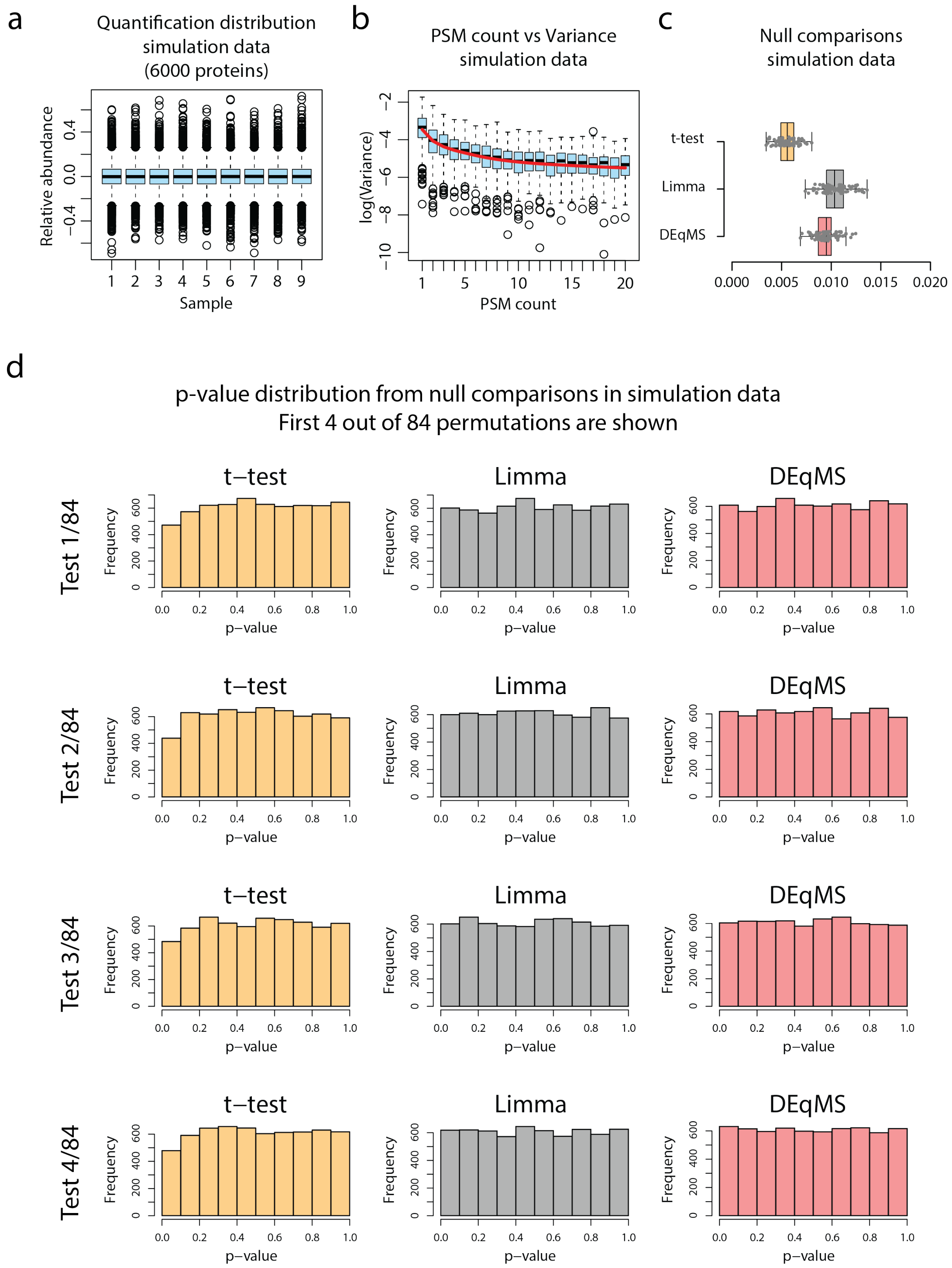

Supplementary Figure 3. Simulation data and null comparisons for evaluation of false positive rates for t-test, Limma and DEqMS. **a.** Boxplot showing distribution of quantitative values across the nine samples in the simulation data. **b.** Protein variance within sample groups in relation to the number of PSMs quantified per protein. Only proteins quantified with up to 20 peptides or PSMs are shown. The red curve is fitted prior variance of the proteins in DEqMS. **c.** Box plot showing the false positive rates for 84 null comparisons in the simulation data, calculated for each comparison as the number of genes with raw p-value (not adjusted) <0.01 divided by total number of proteins tested. **d.** Histograms showing t-test, Limma and DEqMS p-value distributions for the first four (out of 84) null comparisons in the simulation data.

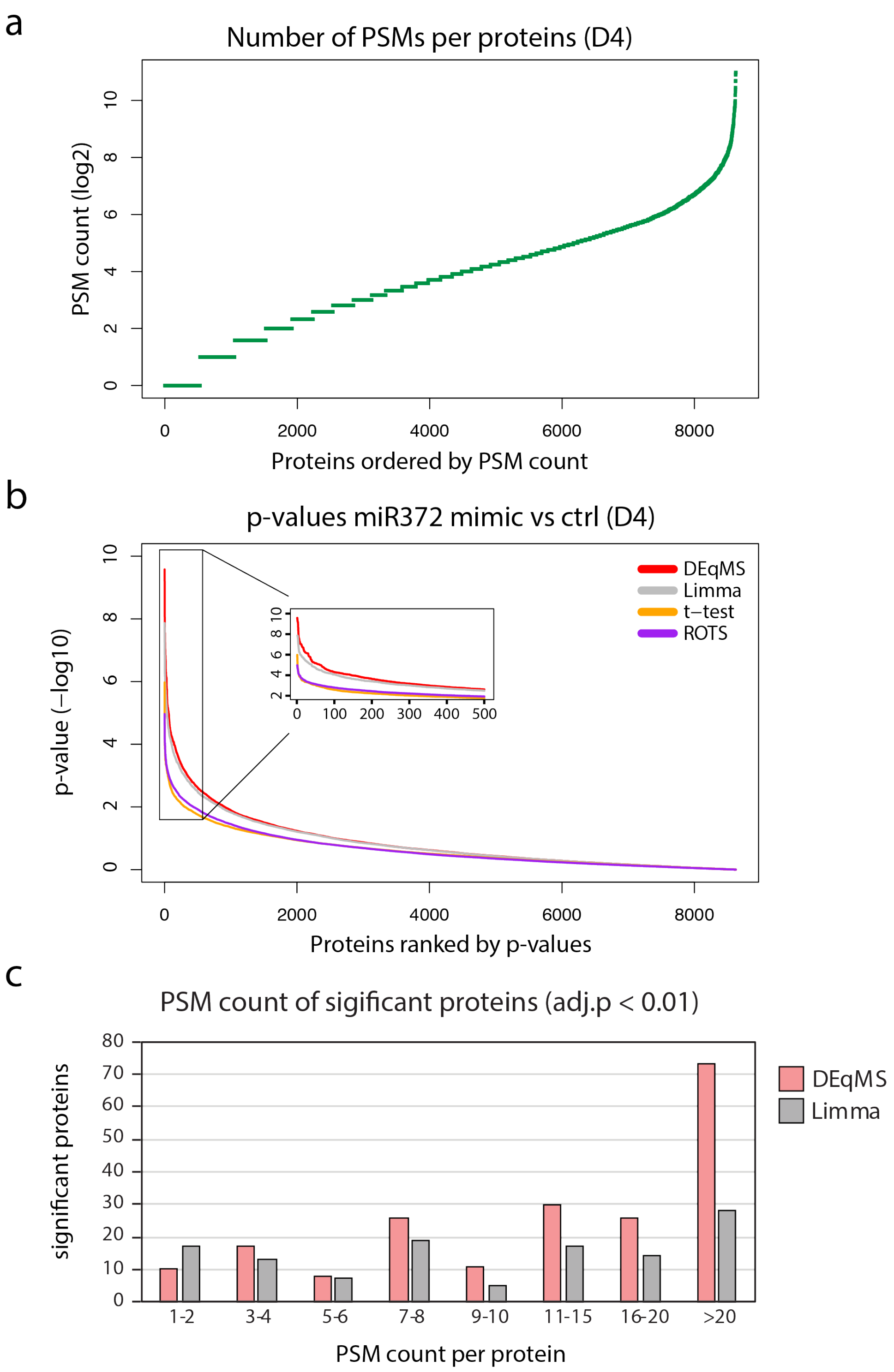

Supplementary Figure 4. Evaluation of DEqMS in a microRNA mimics treatment dataset (D4). **a.** PSM count distribution in dataset D4 showing proteins ranked, from low to high, by number of PSMs per protein used for quantification. Y-axis indicate the number of PSMs per protein used for quantification in log2 scale. **b.** Distribution of p-values (not adjusted) from statistical analysis using different methods for comparing miR-372 mimic treated cells versus control cells. **c.** PSM count distribution of significant proteins (adjusted p-value <0.01) as identified by DEqMS and Limma respectively.

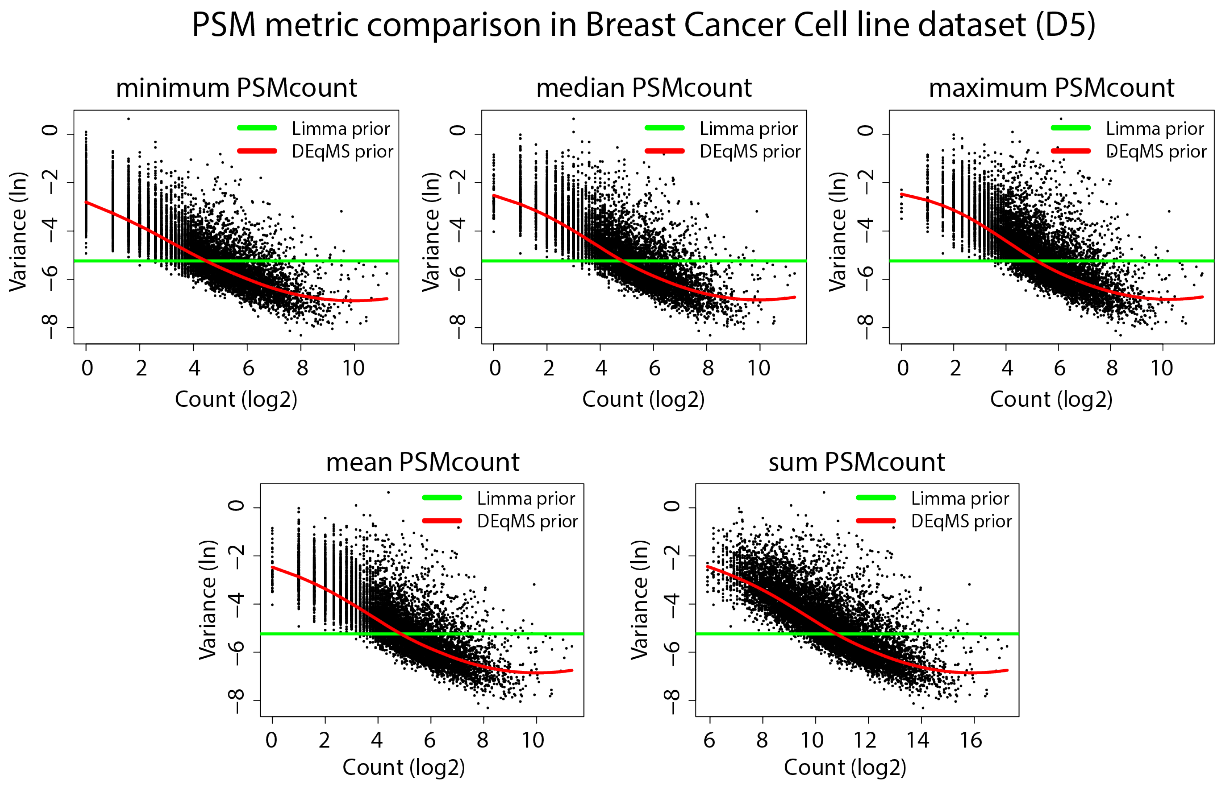

Supplementary Figure 5. Comparison of different PSM count metrics for datasets based on more than one TMT-experiment. Shown in the plots are pooled and prior variances for the Breast cancer cell line dataset (D5) in relation to minimum, median, maximum, mean and sum PSM count. PSM: peptide spectrum matches, (ln): natural logarithm.

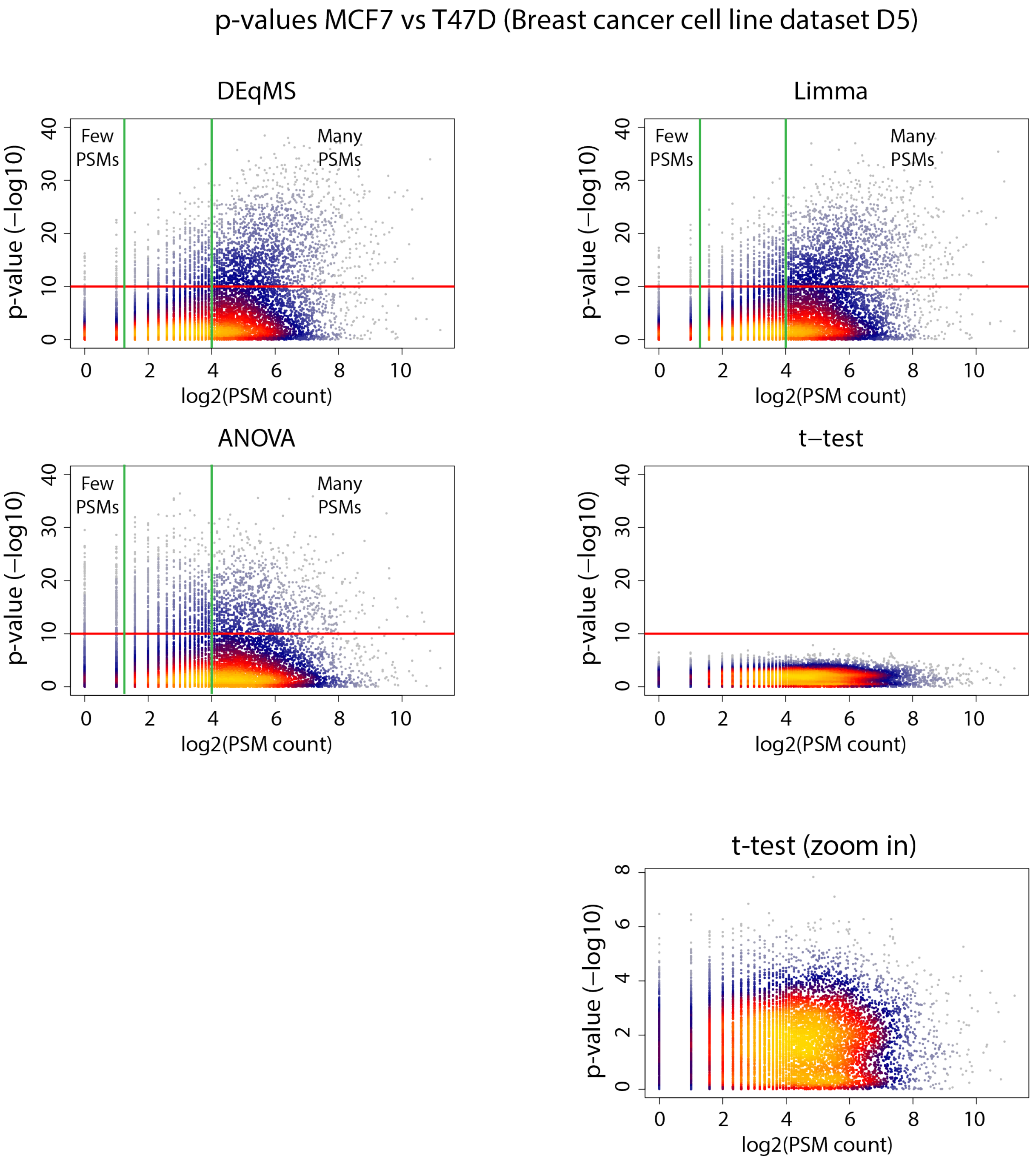

Supplementary Figure 6. Output from DEP analysis comparing the two breast cancer cell lines MCF7 and T47D using different statistical methods. Plots from top to bottom, left to right show P-value distribution in relation to number of PSMs used for quantification for DEqMS, Limma, ANOVA and t-test respectively. For reference purpose, a red line was added at p-value: $1x10^{-10}$. Also shown in the bottom row is a zoom in of the t-test plot. Note the difference in p-values between DEqMS, Limma and ANOVA for proteins quantified by few (1-2) PSMs or many PSMs as defined by the green lines.

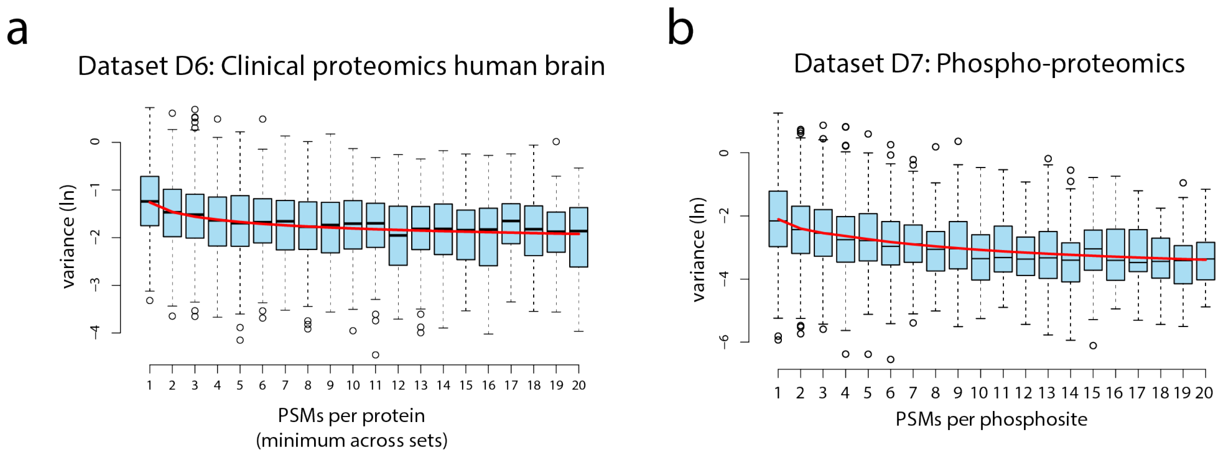

Supplementary Figure 7. Variance within sample groups in a clinical- and a phosho-proteomics dataset. **a.** Boxplot showing the variance in TMT-labelled clinical proteomics experiment (D6) for proteins quantified by 1 to 20 PSMs. **b.** Boxplot showing the variance in a TMT-labelled phospho-proteomics experiment (D7) for phosphosites quantified by 1 to 20 PSMs. PSM: peptide spectrum matches, (ln): natural logarithm.
